# Prevalence and Risk Factors for Suicidal Behaviors Among Medical Students Globally

**DOI:** 10.1192/j.eurpsy.2026.12083

**Published:** 2026-07-31

**Authors:** F. Agyapong-Opoku, N. Agyapong-Opoku, B. Agyapong, A. Greenshaw

**Affiliations:** 1 School of Medicine, University of Galway, Galway, Ireland; 2 Deparment of Psychiatry, University of Alberta, Edmonton, Canada; 3 University of Ghana, Accra, Ghana

## Abstract

**Introduction:**

Suicidal ideation and suicide attempts represent major public health concerns among young adults, particularly those in demanding academic environments. Medical students experience disproportionately high rates compared to their peers in the general population and other disciplines, underscoring the urgent need to address mental health challenges in medical education.

**Objectives:**

This scoping review synthesizes evidence from systematic reviews and meta-analyses on the prevalence and risk factors of suicidal ideation and suicide attempts among medical students worldwide.

**Methods:**

Following the PRISMA-ScR framework, six databases were systematically searched for peer-reviewed reviews published within the past decade. Eligible studies focused exclusively on medical students and reported data on the prevalence and/or risk factors of suicidal ideation or suicide attempts. Twelve reviews met the inclusion criteria, encompassing a combined sample of 378,081 medical students. Data were extracted on prevalence estimates, risk factors, study characteristics, and key recommendations.

**Results:**

Across the included reviews, lifetime prevalence of suicidal ideation ranged from 2.9% to 53.6%, while pooled estimates from meta-analyses ranged between 11% and 25%. Pooled prevalence of suicide attempts ranged from 1.64% to 8%. Depression consistently emerged as the most significant risk factor for both suicidal ideation and attempts. Additional factors included anxiety, burnout, female gender, financial strain, and academic stress. Suicidal ideation was more prevalent during the COVID-19 pandemic and among clinical-phase students. Gender differences in suicide attempts were inconsistent. Overall, medical students exhibited higher rates of suicidal behavior than other university populations.

**Conclusions:**

Suicidal behavior remains a critical and persistent mental health issue among medical students globally. Despite the identification of multiple risk factors, evidence-based interventions are scarce. Future research should emphasize longitudinal and interventional designs to explore causal mechanisms, regional differences, and post-pandemic effects. Strengthening institutional support systems and implementing early prevention strategies are essential to reducing suicide risk in medical education.

**Disclosure of Interest:**

None Declared

